# Editorial: The role of novel hepatitis B biomarkers in solving therapeutic dilemmas

**DOI:** 10.3389/fmed.2023.1256109

**Published:** 2023-07-26

**Authors:** Ivana Lazarevic, Valentina Svicher, Maja Cupic

**Affiliations:** ^1^Institute of Microbiology and Immunology, Faculty of Medicine, University of Belgrade, Belgrade, Serbia; ^2^Department of Biology, University of Rome Tor Vergata, Rome, Italy

**Keywords:** hepatitis B virus (HBV), chronic hepatitis B (CHB), new diagnostic methods, HBV biomarkers, HBcrAg, HBV RNA, HBV cccDNA

The present-day management of chronic hepatitis B virus (HBV) infection depends on persistent monitoring of viral activity, disease progression, and treatment response (1). The goal of therapy for chronic hepatitis B (CHB) is to improve survival by preventing the risk of cirrhosis and end-stage liver disease and to improve quality of life. Unfortunately, a complete (sterilizing) cure is not achievable by current therapies since they do not affect the persistence of viral covalently closed circular DNA (cccDNA) in the liver cells or the viral DNA integrated into the host genome. Thus, the surrogate treatment endpoint of HBV surface antigen (HBsAg) seroclearance has been established, known as a functional cure (2, 3). However, this endpoint is only achieved in the minority of patients, while for the majority, an indefinite length of nucleos(t)ide analogs (NA) therapy is needed.

Traditional biomarkers have proven to be insufficient in resolving the problem of therapy length in the majority of patients as well as in predicting clinical outcomes. Measurement of intrahepatic HBV cccDNA levels and its transcriptional activity is becoming crucial for managing CHB patients and individual therapy approaches (3–5). However, the need for liver biopsy strongly limits the evaluation of cccDNA as a routine practice. In addition, HBsAg presence is not dependent on cccDNA transcription alone because its primary sources are the subviral particles derived from viral DNA integrated into the host genome (3, 4). For these reasons, an urgent need has developed for new non-invasive biomarkers capable of reflecting the intrahepatic activity of the virus and assessing the likelihood of achieving partial or complete functional cure, predicting the risk of liver-related complications and the possibility of viral reactivation in immunosuppressed patients (4–6).

This Research Topic was designed to scope experiences with new viral, immunological, genetic, and epigenetic biomarkers that can contribute to the resolution of current therapeutic and diagnostic dilemmas of HBV infection. It comprises three original research articles, one narrative review, and one meta-analysis.

Although earlier concepts regarding HBV replication did not acknowledge the presence of HBV RNA outside hepatocytes, it is now known that HBV RNA (pregenomic -pgRNA and total RNA derived from variants with defects in RNA splicing) can be detectable in the serum of patients who are either treatment-naïve or treatment-experienced and indicate cccDNA transcriptional activity and reservoir size. Tao et al. investigated the kinetics of serum pgRNA in chronically-infected patients during long-term NA therapy and showed a generally decreasing trend of pgRNA from baseline to 96 months after treatment start. The NAs can block the HBV DNA reverse transcription from the pgRNA and lead to the accumulation of encapsidated pgRNA in the initial period. The study reaffirmed that HBV DNA decreased more quickly than pgRNA in NA-treated patients and that pgRNA can still be found in patients with undetectable HBV DNA. This is important because patients with sustained undetectable HBV DNA but positive serum pgRNA are more likely to experience viral rebound after discontinuation of NA therapy. The study established that low-level serum pgRNA at baseline or significant decline at month 6 may predict the high incidence of undetectable serum pgRNA at year 4 following NA therapy. In addition, they demonstrated that pgRNA may serve as a novel predictor for HBeAg seroconversion during NA therapy.

On the other hand, a different value of HBV RNA was presented in the study by Kaewdech et al.. They investigated the clinical utility of the SCALE-B score to identify the candidate patients who can safely stop NA therapy. The SCALE-B model incorporated quantitative HBsAg, hepatitis B core-related antigen (HBcrAg), age, ALT at the end of treatment, and tenofovir use. This model significantly predicted both viral and clinical rebound and HBsAg loss, while measuring HBV RNA was not associated with off-treatment outcomes. The study confirmed the observation that the combination of different biomarkers has a much higher diagnostic value than the utilization of a single viral biomarker, corroborating the importance of a proper integration of HBV markers in optimizing the management of patients with chronic HBV infection. This is also substantiated in the findings of Thanapirom et al., who evaluated different biomarkers and their combinations to identify the treatment eligibility of patients with CHB. The TREAT-B score, comprising ALT level and HBeAg serostatus, was a simple and accurate alternative to different international guidelines criteria for identification of patient eligibility for HBV treatment.

Among patients who have recovered from acute hepatitis and those who have stable chronic infection, in case of severe immunosuppression, a significant viral replication can be established anew and lead to hepatitis flare and liver failure, i.e., HBV reactivation (7). The review article by Chang et al. brought an extensive overview of the mechanisms and risks of HBV reactivation in various clinical settings. It also presented the utility of new HBV biomarkers in assessing risk and protective factors for HBV reactivation, together with cut-off values for quantitative methods. The viral biomarkers identified as risk factors were HBsAg and HBeAg positivity, high HBV DNA level (>10,000 IU/mL), and high level of anti-HBc antibodies (≥6.41 IU/mL). In contrast, anti-HBs positivity, high anti-HBs level (>56.48 mIU/mL), and HBV genotype A were shown as protective markers.

Finally, the meta-analysis by Ying et al. presented results concerning genetic biomarkers associated with HBV treatment success. The study, comprising 13 studies and 2,510 patients of Asian, African, and Caucasian ethnicities, revealed that the IL-28B rs12979860 CC genotype and rs8099917 TT genotype indicated a better treatment response than non-CC and non-TT genotypes for PEG-IFN-alpha in patients with CHB.

These articles deliver a closer perception of the value of new viral and genetic biomarkers in guiding diagnostic and therapeutic dilemmas. Based on the given results, it is evident that new biomarkers' diagnostic and prognostic power can be improved when they are applied in combinations and according to specific indications.

## Author contributions

All authors listed have made a substantial, direct, and intellectual contribution to the work and approved it for publication.
